# International Medical Graduates' perceptions about residency training experience: a qualitative study

**DOI:** 10.5116/ijme.63c3.e6b3

**Published:** 2023-01-27

**Authors:** Marghalara Rashid, Julie Nguyen, Jessica L. Foulds, Gordana Djordjevic, Sarah E. Forgie

**Affiliations:** 1 Department of Paediatrics, University of Alberta, Edmonton, Alberta, Canada

**Keywords:** IMGs, grounded theory, residents, perspectives

## Abstract

**Objectives:**

To explore International Medical Graduates residents' experiences and perspectives of their residency training program.

**Methods:**

This qualitative study was conducted at a large research-intensive University. Purposeful sampling was used to recruit 14 International medical graduates. The residents recruited for this study were at different levels in their training ranging from Postgraduate year one to five. Residents interviewed represented seven unique specialties. Each trainee was interviewed, and the data were recorded and transcribed verbatim. A thematic analysis framework was used to conduct the data analysis, resulting in the development of study themes.

**Results:**

Our analysis generated six main themes. These themes were related to costly decisions, unspoken expectations, the stigma associated with being an IMG, fears of being an IMG, the strength and resilience of IMGs, and recommen-dations proposed by IMGs for program improvement.

**Conclusions:**

In this study, we wanted to explore international residents' experiences with their programs. The experience of each individual international resident is unique. However, in this study, we were able to provide firsthand perceptions of IMGs from a research-intensive university and identified common themes experienced and perceived by our resi-dents. This study's findings may help educate, reduce stigma, and guide the implementation of effective individu-al and systemic support for these trainees. Which in turn will enhance the overall educational experiences for IMGs trainees. Our study found that themes seem to be recur-ring, hence, an urgency to bring about appropriate chang-es, equitable opportunities, and support for IMGs.

## Introduction

The term International Medical Graduates (IMGs) is used to describe several kinds of medical school graduates. Immigrant IMGs (I-IMGs) are foreign born physicians who have medical degrees from countries outside of North America.^1^ Ninety percent of IMGs are born in other countries and two-thirds of those are from underdeveloped or developing nations.^2^^,^^3^ This data reflects a shift from previously reported data on the origins of IMGs in Canada, who were mostly from European countries.^4^

International graduates play an important role in the medical system filling significant gaps in primary care provision,^5^ in care delivery for vulnerable patients,^6^ and in rural medical care.^7^

IMG residents frequently face discrimination which affects their training and career trajectories.   IMGs are often older, have more experience with complex diseases, and are further away from their undergraduate medical education, raising further concerns of IMGs knowledge and skills.^8^ A qualitative study conducted in 2008 focusing on IMGs found that many faced challenges in their residency programs and experienced three phases of adjustment: loss of professional identity, devaluation and status; confusion around roles and responsibilities; and finally, adaptation and familiarity with the system.^9^ Additionally, internationally trained residents from a wide range of residency programs indicated that they faced issues with acculturation, lack of support, and adjusting to their new programs. Applying for residency programs is a stressful experience for all trainees, but this effect is increased for IMGs.^9^ A literature review conducted in 2012 revealed that IMGs face a wide range of post-migration stressors, including communication problems, culture shock, and discrimination. Once in training, the review noted IMGs were subjected to unfair feedback from preceptors and stress associated with the perception of being "not good enough" as a trainee.^10^  IMGs are also more likely to experience stress compared due to a lack of familiarity with the host medical system and overall healthcare culture.^10^ A recent meta-ethnography identified over 2900 studies pertaining to IMGs, but only 57 studies spanning from 1977 to 2019 were included highlighting the sparseness and punctuated nature of IMG research.^11^ The study conducted by Al-Haddad and colleagues illustrates that some experiences are not exclusive to IMGs within our sample. However, the extent of these issues and concerns, which span across many medical schools, seem to have not changed over time.^11^ Previous empirical research on understanding IMG residents' experiences and perceptions of their training programs makes implementing effective individual and systemic support challenging. Therefore, our objective was to explore IMG residents' experiences of their residency training program at present.

## Methods

### Study design

A qualitative design, based on the principles of naturalistic inquiry, was used to examine IMGs perceptions of their residency programs. This study was guided by an approach to qualitative research known as descriptive qualitative design.^12^ Descriptive qualitative design can be applied across a wide range of ontologies and research questions.^12^ The design was selected because it can be used with a wide range of sampling and data collection approaches, and it aligned appropriately with our study objectives.^13^ By obtaining firsthand information from participants, their responses can be utilized by educators and residency programs to better understand international residents' perspectives and support them in their programs. Our study was approved by our university's Research Ethics Board (REB) and Trainee Research Access Committee (TRAC). All participation was voluntary, and all participants provided informed written consent.

### Sampling and recruitment

Purposeful sampling was used to recruit study participants. We recruited IMG residents from a single Canadian institution, representing all years of training (n=14). Of the 14 participants, seven individuals identified themselves as female, and the remaining^7^ identified themselves as male.   IMG residents interviewed were at different levels in their training (ranging from Postgraduate year (PGY) one to five. IMG residents interviewed represented seven unique specialties, including internal medicine, adult neurology, medical microbiology, pediatrics, psychiatry, radiology, and family medicine. Four of these residents were Canadian born, and 10 were foreign born residents with foreign training. At our center IMG positions are funded through the provincial government and are allocated according to provincial physician resource needs with a capped total number of positions. These positions are divided between family medicine (50%), general specialties (35%, Anesthesiology & Pain Medicine, Internal Medicine, Neurology, Pediatrics, Public Health & Preventive Medicine, & Psychiatry), and non-generalist or laboratory medicine disciplines (15%). The applications are competitive, and eligibility is overseen by a provincial program that coordinates with designated postgraduate residency programs within the province. Once residents are selected and have completed a successful evaluation externship, they integrate into the standard residency programs respectively. A purposeful sampling of our trainees ensures diversity and saturation of varied participant perceptions for ultimate theory development. The number of study participants was not predetermined, and it was dependent on when we reached saturation. Data saturation was the point at which no new information emerged from our interviews and hence, an indication that data collection could be stopped.

### Data collection

This study was conducted during the COVID-19 pandemic when physical distancing measures were in place. Therefore, participants were invited to take part in 40–60-minute telephone interviews with a trained qualitative researcher at a time that was chosen by the participant. An interview guide was developed and piloted for the telephone interview, with numerous probes to get as many details as possible from our study participants. The interviews were not constrained by the interview guide. Our focus was on the participants' conversations, and numerous spontaneous questions were asked during our interviews as well. This allowed us to fully capture our participants' voices and perspectives. Data collection continued until data saturation was achieved.

### Data analysis

Interviews were digitally recorded to facilitate data analysis. Interview data were transcribed and subsequently organized as verbatim transcripts in a table for data management and analysis. Thematic analysis^14^ was used to analyze our data incorporating the following steps: All our team members conducted multiple readings of the transcripts to get familiar with the content of the data (Step 1). As a team, we identified and assigned preliminary codes derived from the transcripts related to our study participants' experiences (Step 2). Following this preliminary analysis, we then carried out an in-depth analysis of the data based on the highlighted codes. In this phase of the data analysis, the preliminary codes that were identified in Step 2 were discussed and created a basic thematic schema. In this stage, we actively engaged in evaluating if codes were strong enough to be included or excluded within the emerging thematic categories (Step 3). We continued the process of reviewing themes in our data analysis meetings, which gave us time to reflect and further review the themes as a group on multiple occasions (Step 4). Further review and discussions occurred in the follow-up meetings, where the focus was on defining and naming our themes to effectively represent our data (Step 5). Finally, our goals were to disseminate our study results in conferences and as a manuscript in academic journals (Step 6).

### Quality and trustworthiness

To ensure quality and rigour, including accomplished researchers, correct methodology and disciplined methods. We used peer debriefing, memos and field notes for research quality.  Peer debriefing: Research teams conducted regular meetings to review transcribed data and make decisions on recruitment and overall study progress. Team members provided feedback on the conceptualization of the core phenomena to ensure congruence existed between the collected data and our analysis. Through this we ensured any personal bias of the researcher(s) did not influence the analysis of the participant responses.^15^^,^^16^ Memoing and field notes: Diligent memoing and field notes enhanced the rigour of the study as it included the thoughts, feelings, and innate contemplation of the researchers. The memos served as an audit trail for the creation of categories and interrelationships between categories.^17^^,^^18^

## Results

The data revealed six themes: Costly decisions, unspoken expectations, the stigma associated with being an IMG, fears of being an IMG, strength and resilience of IMGs, and recommendations proposed by IMGs for program improvement.

### Costly decisions

The majority of the study participants reported that the cost of exams and the application process for applying to a residency is program was extremely expensive, as evident in the following statements:

"To be honest, I didn't take a lot of courses because I just didn't have enough money. A lot of people spend a lot of money to take those courses for preparation for those exams as well as the English language [test]. But easily, I spent probably around $15,000, easily, just going through all those four exams and the whole application process and the travel and everything that is associated with that. That might not be financially viable." [Interview 10, Male, Internal Medicine]

Our study participants also spoke about costs related to raising a family, as IMGs often are applying at different life stages, and the financial responsibilities as demonstrated in the following quotation:

"…it was very, very hard with two kids just to manage, first of all, enough time to do a job to earn enough to live on top of that save enough money to take all those exams, and everything costs money, from the application to [The Alberta International Medical Graduate] AIMGP to everything, to [International English Language Testing System] IELTS and everything." [Interview 10, Male, Internal Medicine]

Additionally, residents experienced a tremendous amount of pressure related to the ambiguity associated with getting into the program and the time needed to obtain the income to pay for the preparation and the exams:

"I think the loans and the ask to borrow money would have been too great, and it's a lot of pressure if you don't match after you finish medical school. I know quite a number of foreign IMGs who came to Canada would have to do a job that they didn't want to do. An example would be security at night or something like that so they could retrain and try and get into the system, but then they would be in debt. They would have to find another job. It would take them years to accrue enough money to write the licensing exams." [Interview 2, Male, Medical Microbiology]

### Unspoken expectations

Participants reported that there were certain expectations from IMGs residents that were similar to their counterparts, but the expectation to traverse the learning gap was a struggle. Participants reported initial struggles in navigating to get familiar with their hospital culture or getting familiar with the system. As reported by a participant's statements:

"Over here, third, fourth year medical students are given a lot of autonomy, they can co-sign. With a co-sign, they can write orders. They can get involved and assist with surgeries even. And I think that in the UK, you're still very much an observer, [here] there's a lot of independence expected right away …[the]electronic system, which is not necessarily the case in all parts of UK that might have changed more recently, but that was something I had to get used to as well. So basically, getting used to a new hospital system, a new culture in which how things are run and the level of autonomy that's given to trainees." [Interview 1, Female Neurology]

In order for IMG trainees to meet these expectations they felt that they had to work harder than CMEs as evident in the following statement:

"I felt like sometimes I worked a lot harder than they did. I would do discharges at 7:00 p.m. in the evening when they were done at 12:00 p.m. and got home and doing whatever. Or I would have my discharge summaries ready the day before the person was discharged. So they are ready and would have an efficient day to not waste time on updating discharges. I would do so many extra things just to be efficient, be a good resident." [Interview 7, Female, Psychiatry]

### Stigma associated with being an IMG

 Many participants reported stigma associated with being an IMG. They reported feelings of being an imposter, systematic stigmatization, and they felt their international medical training and educational past may be looked down upon. Stigmatization of IMGs is usually associated with beliefs that international medical schools are perceived as being inferior. In addition to the discrimination based on international medical curricula, accents also create an othering effect as evident in the participant statement below:

"I think there are some Canadian grads out there who may look down on IMGs, both Canadian grads because there's this assumption that if you couldn't get into a Canadian school and you had to go elsewhere……. And this misconception that because this person has an accent, they won't understand what you're saying, or they don't understand the system. I think there's no education to physicians, residents, and medical students who were trained in Canada to identify what a foreign international medical grad goes through to get to where they're at." [Interview 2, Male, Medical Microbiology]

Many IMGs felt they were encountering biases that prevented their assessment from being utilized to their fullest abilities and their clinical skills that they bring to patient care. Ongoing stigmatization of education thresholds may cause physicians to underestimate IMGs regardless of their performance as stated by a participant below:

"For myself. In some of my placements. As you go from rotation to rotation, I had one where the people knew me from before, years before medical school. All those doctors because I had worked with them as a paramedic. And they knew where I went to medical school, and I think that they may have had a particularly jaded opinion of me from the outset, partly- in large part because of the school I went to." [Interview 3, Male, Family Medicine]

### Fears of being an IMG

IMGs describe elements of fear and anxiety associated with failure, judgement as an IMG, and reluctance to speak out: 

"I think that's just an innate insecurity, anxiety on my part. But I can only guess that I'm probably not the only person." [Interview 3, Male, Family Medicine]

In addition to experiencing personal fears, external factors such as reactions from peers, colleagues, and mentors can lead to uneasiness:

"When you're brushing shoulders with them, there's a sense of inferiority of the fact that, oh, they got in, but we didn't, and we had to go overseas." [Interview 5, Male, Pediatrics]

Lastly, IMGs may be afraid to speak up or speak out due to the perception that they should be grateful for the opportunities and may often be misunderstood because of these fears:

"But the sad reality is that we don't have a strong voice, and everybody's too afraid to speak up against that because you don't want to be labeled. You don't want to be red tagged. You don't want to be red flagged. Then just because there's too many of us trying to get into the system, a lot of us have kind of [said], "Okay." Even if there is some discrimination, we are still okay to accept that because you just really want to get into the system." [Interview 10, Male, Pediatrics]

### Strength and resilience of IMGs

Many of the study participants acknowledged the hurdles to become a practicing physician and the amount of training needed to meet healthcare standards in their new home country. However, many participants alluded to the strengths and benefits the foreign healthcare system experiences when including IMGs. For example, the diversity of knowledge and experiences as portrayed in the following quote:

"I think we also bring cultural experiences as well, which is acknowledged not just by the medical team and the program, but also by the patients. Seeing that Canada is a multicultural country, to see this also in the physicians treating them and the nurses treating them, in the allied health treating them gives a bit of reassurance and builds that trust and rapport, and it depends on the level of training. IMGs are also very valuable in their own strengths. When you talk to them, it's fairly unique." [Interview 11, Male, Pediatrics]

In addition to contributing to the diversity of medicine, IMGs are resilient, continuing with the rigorous application process as stated in the following statements:

"Recognize that IMGs don't just arrive in Canada and get residency spots. They must be vetted the same way, if not more rigorously than Canadian medical students. We must write a slew of exams before we are even allowed to apply to CaRMS [Canadian Residency Matching Service]. Once we apply to CaRMS, we go through the same application process, the same interviews, so the vetting is very, very rigorous, and to be aware of that, so maybe some knowledge or faculty development on what the IMG process is would be helpful to reduce bias." [Interview 4, Female, Internal Medicine]

"I believe residency is overwhelming. It's a lot of work, long hours, but I think the journey that we've all gone through, especially myself to reach to this point has been more over resilient ... We had to show more resilience rather than residency itself. I feel like now that I'm in residency, I feel like this is what I've worked so hard for, so I need to do a good job and I don't feel the burden of, oh, I have to work 24, 25 hours today." [Interview 13, Female, Internal Medicine]

### Recommendations proposed by IMGs for program improvement

Our participants gave both jarring and wonderful insights on the obstacles international medical graduates may encounter in their attempt to gain training and subsequent licensing in Canada. Each resident provided ideas and recommendations to reduce some of the barriers and stressors. For example, creating a mentorship program specifically with previous IMGs would allow current IMG trainees to create connections within their program and with staff that have gone through a similar process, effectively establishing a more supportive community:

"I certainly recommend having a mentorship program, when somebody that is an IMG, that is in residency, would be great if they can have a mentor during the first six months. So a mentor could be either another staff, an IMG staff that is already done with the process or a resident that has an IMG background too. Because that will help to kind of go through this process." [Interview 8, Female, Internal Medicine]

As an extension of the mentorship program, other IMGs have suggested creating a stronger, more active association for IMGs allowing them to have a more unified and effective voice. A concern previously brought up by IMGs was the fear of individual repercussions, but an established, recognized association would allow for the representation of the individual.

"I just don't think that we have a strong voice as an IMG cohort throughout Canada. We don't have an association of some sort which is unified. It's like supply and demand almost kind of thing, that there's too many IMGs who are trying to get in the system for too little spots. And people can get away with doing whatever they want because nobody's going to complain. And if you are the one who is complaining, just step aside. There's lots of people behind you in the line who's ready to take up on any offer that they're offered. Right? Something is better than nothing, almost." [Interview 10, Male, Internal Medicine]

Becoming an IMG within the foreign medical system is incredibly difficult, and our participants felt that many foreign trainees and staff have no idea about the prejudices and other struggles they face. Increasing awareness about the difficulties of becoming an IMG would hopefully create empathy and create a better learning environment and foster mutual understanding and respect.

"I think just creating awareness of how difficult it is to get where we get. Again, I think there's value to have that diversity in the Canadian healthcare system, but I think we just need as you keep people, you know do not be prejudice against IMGs. Again, that doesn't make them worse or less important because again, they bring a lot of value in my opinion. Honestly, the IMG that I know, they're probably the most hard-working people that I know in this residency. I think people should be educated about it." [Interview 7, Female, Psychiatry]

## Discussion

This study included international medical residents' perceptions from a broad range of postgraduate programs at our diverse, urban post-secondary institution. As such our findings are likely reflective of experiences of other residents in similar size centres. Through the use of a qualitative design, we were able to gain an in-depth knowledge about IMGs perspectives of their residency programs. Our analysis generated six main themes. These themes were related to costs associated with the entrance exams and the application process to residency programs, unrealistic expectations, stigma and fear, ideas for improvement, and the strengths that IMGs offer to the training environment and workforce.

Based on Al-Haddad and colleagues' study in (2019)^11^ similar themes regarding IMGs experiences have arisen. Examples included challenges that are associated with immigrating, such as recertification and settling in, stigmatization of IMGs in developed countries, and the benefits of IMGs and their resilience. The overlapping nature of themes highlights issues that have sadly plagued the medical systems across the world for decades (1977- 2019), highlighting pervasive experiences, needs and a critical gap in learner support as evident in our current findings. Perhaps most poignant from our work was the theme of stigma and fear. Stigma and discrimination against IMGs have been reported previously.^19^ A qualitative study conducted in 2006 by Woods and colleagues^20^ suggested that their study participants experienced considerable discrimination and prejudice as IMGs in the United States. Similarly, a Canadian study conducted in 2015, revealed that international residents in family medicine reported being singled out, rejected, and mistreated simply based on their status as an IMG.^21^ Our findings add further evidence from several disciplines at our centre highlighting the discrimination experiences of IMGs which has persisted over decades.

IMGs encounter numerous issues related to acculturation and stigma; it is estimated that approximately 30% of IMGs face discrimination in their new home country.^22^^,^^23^ Despite the fact that IMGs bring diversity, a wide range of unique experiences, and medical knowledge, there is both subtle and more overt stigma that was experienced by our study participants. Methods to report, address, and prevent microaggressions are essential for programs to establish in order to provide a psychologically safe learning environment. As Trinh and colleagues noted in their narrative review of cultural competence and humility in psychiatry education, cultural humility refers to the "ability to maintain an interpersonal stance that is open in relation to the cultural aspects of cultural identity that are most important to the patient."^24^  Our participants note opportunities for postgraduate programs to foster cultural humility, and encourage that "open" interpersonal stance that acknowledges and  works toward alignment with our local institution's vision statement of its Strategic Plan for Equity, Diversity and Inclusivity Plan: commitment "to cultivating an institutional culture that values, supports, and promotes equity, human rights, respect, and accountability among faculty, staff, and students."^25^ Our participants, perhaps not surprisingly by their willingness to participate in this study, were motivated to help with this process and  contribute to reducing stigma and calling out discrimination. This, however, can come as an increased pressure when there are increased perceived pressures to perform and meet expectations, let alone add more responsibilities to help foster diversity on minority trainees: also known as the minority tax.^26^ IMGs are educational minorities within many foreign postgraduate training programs, and we must be mindful that may also be underrepresented minorities. The impact race/ethnicity as well as educational background that may impact their experience, and experience of discrimination.

Discrimination was perceived by both Canadians who studied abroad (C-IMGs) and foreign-born/international IMGs (I-IMGs). Canadians who studied abroad expressed feeling judged for not entering Canadian undergraduate medical programs which added to their own feelings of being an imposter. A recent multiple case study of Canadian and I-IMGs from a large Canadian centre, representing family medicine, internal medicine, and surgery, compared experiences of C-IMGs and I-IMGs where C-IMGs expressed that they were "almost like a hidden minority", aware of the Canadian culture but shocked by the transition and learning of a new healthcare system.^27^ Our C-IMG participants shared similar experiences. Interestingly our C-IMG participants shared less concern about their provincial orientation process being skewed to support for I-IMGs, and a more shared perspective with all our participants was that there was a lack of appreciation or awareness of the hurdles and process they have faced. Faculty and resident development to share and sensitize to these issues, as part of an overarching equity, diversity and inclusion curriculum that respects and draws upon differences are important next steps from this work. These results are invaluable for curriculum development and reflective practice for medical educators. Prior work by Zulla and colleagues^28^ demonstrated significant variability between self-rated perceived needs of IMGs by program directors and IMGs themselves. IMGs within residency training are diverse and their experiences heterogenous; considering the impact of cultural differences on individual professional identity formation and how this informs cross-cultural feedback and communication is essential.^29^ Discrimination is not unique to trainees, with particular attention recently being drawn to Islamophobia within the larger medical community,^30^ however learners are in a particular vulnerable position with their feedback and evaluations.

### Limitations and strengths

There are limitations to qualitative research, such as our small sample size and potential lack of transferability. The research was conducted with a sample population at a single academic institution and therefore we reserve transferability. Limitations aside, the use of a descriptive qualitative design with observational data is relatively unique. Findings from this study address the lack of observational data in the IMG literature. Additionally, our study participants are from a wider range of specialities that are currently lacking in the existing studies.

## Conclusions

We identified common themes that focused on the participants’ strengths, eagerness to contribute to an improved experience/educational process, the financial costs, the fear, the stigma, and discrimination associated with being an IMG. Regardless of the context, reflecting on these themes may help challenge one's own beliefs. This study provides valuable firsthand perceptions of IMGs experiences which will better inform training programs and identify areas to enhance the overall educational experience for IMG trainees. In addition, this knowledge will help inform and impact future IMGs' experiences and has implemented effective individual and systemic support for these trainees. Sadly, these trends have not changed much since previous work, highlighting pervasive experiences, needs, and a critical gap in learner support. Hence, there is an urgency to bring about appropriate changes, equitable opportunities, and support for IMGs.

### Conflict of Interest

The authors declare that they have no conflict of interest.
